# Stress and strain among veterinarians: a scoping review

**DOI:** 10.1186/s13620-022-00220-x

**Published:** 2022-06-21

**Authors:** Robert Pohl, Julia Botscharow, Irina Böckelmann, Beatrice Thielmann

**Affiliations:** Department of Occupational Medicine, Otto-Von-Guericke University Magdeburg, Magdeburg, Germany

**Keywords:** Scoping review, Veterinarians, Psychological stress, Mental wellbeing, Workload, Stress, Depression, Anxiety, Suicide

## Abstract

The aim of this review is to systematically review studies on work-related stress that may affect the mental health of veterinarians. Studies have indicated a high prevalence of various risk factors for mental disorders among practicing veterinarians. In addition to a high risk of suicide, there is increasing evidence of burnout and depression. A scoping review was conducted using the PubMed, MEDLINE, Scopus, Cochrane Library, Web of Science, PubPsych and PSYNDEX databases. Twenty-one studies (plus seven studies with nonstandardized questionnaires) published between 2000 and 2021 were found that presented data on the effect of workload on the mental wellbeing of veterinarians. All of the included studies indicate a high prevalence of psychological stressors in veterinary practice. The risks of burnout, anxiety and depressive disorders are higher in this occupational group than in the general population and other occupational groups. Subjectively, female veterinarians perceive their psychological workload to be higher than that of their male counterparts. Working hours and ethical dilemmas stand out as major sources of stress. There is a need to improve overall psychological wellbeing of veterinarians. Organizational support services and developing personal strategies for coping with work-related stress can prove helpful.

## Background

The identification of work-related stressors and their effects on health has been gaining in importance for many years among employers and researchers. In a meta-analysis [1] of workplace stressors and the relationship between mortality and health care costs in the United States, which included a total of 228 studies, the major workplace stressors identified were feelings of job inequity, pressure to perform, shift work, feeling of loss of control, low social support in the workplace, and overtime. The study describes that long working hours increase mortality by almost 20%. Moreover, the association between workplace stressors and health is strong in many instances. For example, work–family conflict increases the odds of self-reported poor physical health by about 90% [1]. All stressors mentioned have an impact on the psychological level and are associated with depression, anxiety disorders, and dementia [2, 3]. These stressors also occur among health care workers. In this occupational group, the workloads and their effects on employees appear particularly serious. A large number of studies have shown correlations between job satisfaction and quality of life, physical health, and mental health, especially among health care workers [4, 5]. Primarily, doctors of human medicine in emergency and rescue services and surgery reported high level of subjective stress [6] as well as increased stress-related physiological strain factors [6–8].

Veterinarians are also exposed to similar working conditions and comparable demands. In addition to obvious high physical workloads, which are particularly prevalent in large animal/farm practice [9, 10], psychological stress factors have been examined among veterinarians. Sources of stress identified as psychological stressors in the veterinary profession include work schedules, financial issues, client demands/expectations [11], and ethical dilemmas regarding treatment options [12]. In 2010, Platt and colleagues found in a systematic review that 14 of the 15 studies reported a higher risk of suicide of veterinarians than in comparison groups [11]. Findings indicating a high risk of suicide among veterinarians have also been reported in Germany [13].

Burnout and general occupational stress among practicing veterinarians have been important topics in the veterinary community for many years. Stress in the veterinary profession was reported in Germany as early as 1963 [14], and there have been increasing contributions to this topic in the 1980s and 1990s [15–17]. Since the early 2000s, there has been an increase in the number of international research studies on the professional situation among veterinarians [18–21]. This demonstrates the increasing amount of interest within the research community as well as the need to summarize the findings of these studies to provide information about risk factors (anxiety, ethical conflicts, exhaustion) for occupational mental disorders with an above average duration of illness.

The aim of this scoping review is to provide an overview of the existing evidence on work stress and its effects on the mental health among veterinarians to complement the existing systematic reviews on suicidality [11, 22, 23] or mental disorders in this professional group [11, 24]. Mental disorder is described as a psychological pattern characterised by suffering of the affected person, impairment in one or more important areas of functioning, increased risk of death, or significant loss of autonomy [25]. Whilst the studies included have used various methodologies, our scoping review aims to summarise the prevalence of existing risk factors for threat to mental health in veterinarians.The manuscript is primarily aimed at practicing veterinarians but also includes topics on the general working situation in veterinary medicine. Therefore, veterinary students and employees in veterinary practices can also benefit from the studies and study results described here. The complex topic of mental health should also enable external stakeholders (e.g., social support, occupational scientists, occupational physicians) to gain insights into the profession of veterinary medicine to experience sensitization in dealing with stress and strain among veterinarians.

## Methodology

This scoping review aims to provide an overview of studies related to psychological workload and potential health effects (depression, burnout) in veterinarians. The scoping review was conducted according to a predefined protocol and followed the Preferred Reporting Items for Systematic reviews and Meta-Analyses extension for Scoping Reviews (PRISMA-ScR) guidelines [26]. The protocol was registered in the Open Science Framework [27].

## Search strategies

The PubMed, PubPsych, Scopus, Ovid, Cochrane Library, Web of Science, PSYNDEX, and PsycINFO electronic databases were searched for literature up to July 25, 2021, and the reference lists of included studies were also manually searched to identify additional eligible studies.

The search terms were as follows: »Veterinary emergency service« OR »veterinary physician« OR »veterinary professionals« OR »veterinary surgeon« OR »veterinary physician« OR »Veterinary engineer« OR »veterinary practice« AND »load« OR »stress« OR »strain« OR »work« OR »job« OR »occupational« OR »work load« OR »work stress« OR »mental stress« OR »stress perception« OR »physiological stress« OR »wellbeing» OR »psychological stress« OR »burnout« OR »psyche« OR »mental health« OR »mental work load« OR »dissatisfaction« OR »strain indicator« OR »mental disorder« OR »mental problems« OR »mental illness» OR »anxiety« OR »addictive behaviour« OR »alcohol addiction« OR »suicide«.

To ensure our search was as diverse as possible, search terms such as “addictive behavior” and “alcohol addiction” were also used. Addictive substances are often associated with psychological stress and can provide clues to existing mental illnesses or mental disorders [28, 29]. The inclusion criteria were as follows: practicing veterinarians (regardless of experience level or specialty), original papers, full texts in English or German, and studies with surveys conducted no earlier than 01/01/2000.

**Fig. 1:**
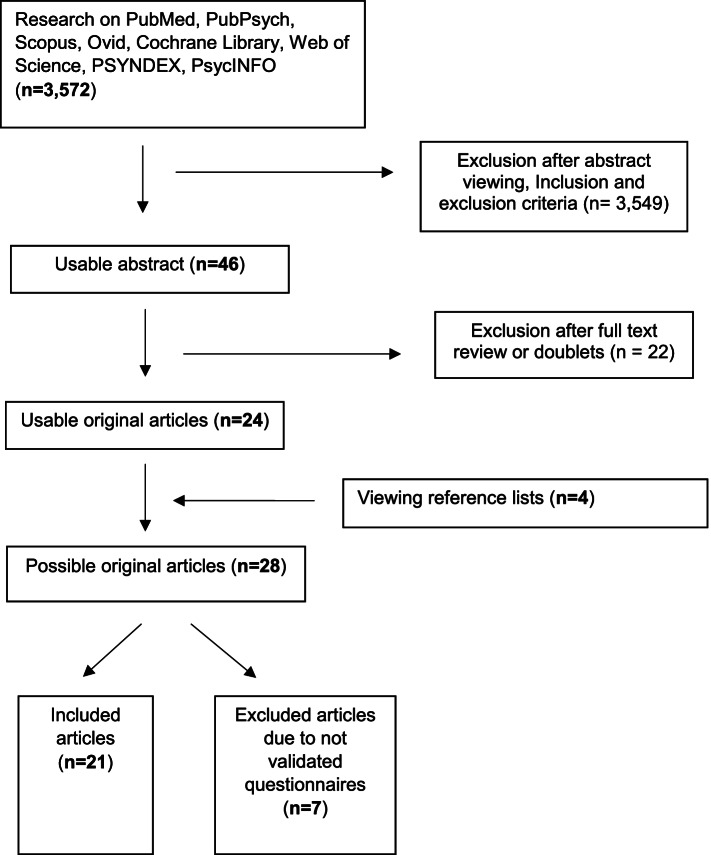
Shows the methodical procedure, including the search strategy, in a flow diagram

Citations not available in open access or that could not be obtained via remote access from the university library or via the authors were excluded. A total of 3,572 articles were retrieved from the literature search (Fig. 1). A total of 4 additional articles was added from the manual search leading to a total of *N* = 3,576. The identified articles were transferred and documented in Citavi 6 Reference Manager (Swiss Academic Software, Wädenswil, Switzerland). Duplicates were manually removed in advance. Two reviewers (R.P. and B.T.) independently screened the titles and abstracts on the basis of the established inclusion and exclusion criteria. In doing so, the articles reviewed were categorized as “suitable”, “other on topic”, and “not suitable”. If there were discrepancies in the categorization of potentially suitable articles, a third reviewer (I.B.) was consulted.

A total of 46 references met the inclusion criteria and were subjected to full-text screening. This screening was performed by the same reviewers. If studies were excluded during the full-text screening, they were discussed by the review team until a consensus decision was reached. Twenty-eight publications remained after the full-text screening, and the overall study quality of these articles was then assessed. Of these 28 studies, 7 additional studies without valid survey instruments were removed, resulting in a total study count of 21, including 7 publications that were requested through the university library or through the authors, as appropriate Fig 1.

## Quality assessment (EPHPP)

The validated Effective Public Health Practice Project (EPHPP) tool [30] was used to assess the quality of the studies. The EPHPP tool is based on six categories (selection bias, study design, confounders, blinding, data collection, withdrawals and dropouts). Each of these items was rated as strong (1), moderate (2), or weak (3) depending on how well the criteria were met. Studies that had two or more “weak” categories were rated weak (3) overall.

## Results

The present review included only studies with valid survey instruments (*N* = 21). Nevertheless, studies (*N* = 7) were found to thematically fit our scoping review but collected data exclusively with self-designed questionnaires [12, 31–35]; thus, these studies were excluded. All of the included studies collected data on sociodemographic factors. Eighteen studies were also differentiated by practice type (small animal, large animal, and mixed practice). The results presented herein consist of 20 cross-sectional studies and one longitudinal cohort study. Table 1 shows the studies with standardized questionnaires found in the full-text search, some of which used supplementary questions (e.g., [36–38]). In addition to the concise results, Table 1 includes details of the study design and sample, as well as the authors and year of publication. In the following text, a part of the important published results with the questionnaires used is described.

**Table 1:** Listing of the studies found according to full text search, indicating the standardised and non-standardised questionnaires, the EPHPP ranking and a selection of main results

First author, year of publication and location	EPHPP raking	Used questionnaire	Sample and study type	Results
Bartram et al. 2009, UK [22]	weak	The hospital anxiety and depression scale (HADS), Three questions on suicidal ideation derived from the second National Survey of Psychiatric Morbidity, The Warwick-Edinburgh mental wellbeing scale (WEMWBS), The Health and Safety Executive management standards indicator tool (HSE MSIT), Subscales of the Survey Work-home Interaction Nijmegen (SWING), A series of 27 original items specifically focusing on potential sources of stress in the veterinary profession (a domain of 9 items referred to clinical work and was only completed by respondents to whom this domain was relevant, An open question inviting respondents to identify in free text up to three main sources of pleasure and/or satisfaction in practice	A sample consisting of 1796 practicing veterinary surgeons (50% male, 50% female) in a cross-sectional study	HADS-A (total mean): 7.9 (± 4.1); non-case (0–7%): 48.8; possible case (8–10%): 25.3; probable case (≥ 11): 26.3% HADS-D (total mean): 4.6 (± 3.4); anxiety subscale non-case (0–7%): 80.6; anxiety subscale possible case (8–10%): 13.6; anxiety subscale probable case (≥ 11): 5.8% HADS-T (total mean): 12.6 (± 6.8) Twelve-month prevalence of suicidal ideation: life was not worth living (23.0%; death wishes (15.0%); suicidal thoughts (21.3%); any suicidal ideation (29.4%) WEMBS (mean score): 48.85 (± 9.06); The mean scores for veterinary surgeons working in university-based non-clinical university-based clinical roles vs. working in small animal practice after adjusting for age and gender (university-non-clinical: β = 3.50, 95% CI: 0.37–6.62, *p* = 0.029; university-clinical: β = 2.76, 95% CI: 0.48–5.03, *p* = 0.018) HSE MSIT: Total mean (95% CI): demands 2.96 (± 0.70), control 3.47 (± 0.78), managerial support 3.14 (± 0.89), peer support 3.75 (± 0.73), relationships 4.01 (± 0.69), role 4.21 (± 0.63), change 3.22 (± 0.94) WHI_N: total mean: 1.19 (± 0.57) WHI_P: total mean: 0.97 (± 0.56) Contributors to stress: Number of hours (42.9%), making professional mistakes (40.4%), client expectations: (38.0%), administrative and clerical tasks (27.9%) Sources of satisfaction: good clinical outcomes (41.5%), relationships with colleagues (33.7%), intellectual challenge/learning (32.4%)
Best et al. 2020, Canada [39]	weak	HADS, MBI-Human Services Scale (MBI-HSS), Professional Quality of Life (version 5) (ProQOL), Connor-Davidson Resilience Scale (CD-RISC)	A sample consisting of 412 veterinary (Nearly 70% of participants identified as female and 30.5% as male) in a cross-sectional study	HADS-A (total mean): 8.8 (± 4.3); non-case (0–7%): 38.7; possible case (8–10%): 28.7%; probable case (≥ 11): 32.6% HADS-D (total mean): 5.2 (± 3.9); non-case (0–7%): 75.4%; possible case (8–10%): 15.6%; probable case (≥ 11): 9.0% Comorbidity defined as both anxiety and depression subscales having scores ≥ 11: 7.1% (4.8–10.0 CI) Approximately 1/3 of participants were classified as probable cases of anxiety based on a HADS-A score ≥ 11, and 9% of participants were classified as probable cases of depression based on a HADS-D score of ≥ 11. Furthermore. Approximately 7% were classified as having comorbid anxiety and depression. Female participants tended to have higher anxiety and depression scores and percentage probable caseness than males MBI-HSS: 36.9% of participants in this survey could be classified as experiencing burnout (95% CI: 32.1% to 42.0%). Female participants (37.8%) tended to have a higher proportion experiencing burnout (95% CI: 31.8% to 44.0%) than male participants (32.7%, 95% CI: 24.2% to 42.2%) ProQOL: Female participants tended to have higher scores in Burnout (44.8% vs. 38.9%) and Secondary Traumatic Stress (72.9% vs. 60.7%), and were more likely to be within the “high” category for these components of compassion fatigue. Compassion Satisfaction: male 36.3% vs. female 31.4%) CD-RISC: The mean CD-RISC score (*n* = 368) was 70.4 (± 14.9). Approximately 74.5% of participants (95% CI: 69.7% to 78.8%) had scores lower than the scale’s comparative norm (the general population of the United States) of 80.7
Cevizci et al. 2014, Turkey [40]	weak	Swedish Demand-Control-Support Questionnaire (DCSQ), self-reported physical and mental health problems by veterinary surgeons	A sample consisting of 223 veterinary (71,7% male, 28.3% female) in a cross-sectional study	DCSQ (mean values between vets in civil servant vs. vets in private sector employee): work load 9.65 (± 1.73) vs 9.26 (± 1.94)*, work control 10.73 (± 2.94) vs. 9.95 (± 2.51)*, skill use 7.12 (± 1.92) vs. 7.02 (± 1.68)*, decision latitude 3.61 (± 1.84) vs. 2.94 (± 1.42)**, social support 11.46 (± 3.58) vs. 11.12 (± 4.19)*. *p < 0.05, **p = 0.008 Reported mental health problems by veterinary surgeons: Unresponded (21.4%), stress (19.7%), short temper (15.4%), Depression (12.8%), Burnout (12.0%), Unhappiness/restlessness (10.3%), Chronic fatigue/insomnia (7.7%), Attention deficit (0.9%)
Crane et al. 2015, Australia [41]	weak	21-item Depression, Anxiety, Stress Scale (DASS-21), Positive and Negative Affect Scale (PANAS), Brief Resilience Scale, Stressor events were identified via three focus groups held with 11 veterinarians (9-point scale), 24-item version of the Multidimensional Perfectionism Scale (FMPS-Reduced)	A sample consisting of 540 veterinary (64.2% female) in a cross-sectional study	inability to pay (*n* = 530): stressor frequency 6.32 (± 2.03) and degree moral significance 59.84 (± 28.40); balancing client and patient welfare (*n* = 501): stressor frequency 5.23 (± 2.05) and moral significance 67.73 (± 25.18); carrying out wishes even if they´re not in the best interest of the animal patient (*n* = 455): stressor frequency 4.63 (± 1.91) and moral significance 74.19 (± 23.78); euthanasia for reasons you did not agree with (*n* = 441): stressor frequency 3.63 (± 1.60) and moral significance 80.04 (± 23.79); euthanasia (*n* = 429): stressor frequency 7.41 (± 1.54) and moral significance 60.08 (± 33.77); assisting with incompetent care (*n* = 353): stressor frequency 3.85 (± 2.03) and moral significance 80.46 (± 22.50); suspected patient/pet abuse (*n* = 131): stressor frequency 2.45 (± 1.43) and moral significance 84.23 (± 20.46) The perfectionism was positively related to stress (*r* = 0.509, *p* < 0.01), anxiety (*r* = 0.473, *p* < 0.01), and negative affect (*r* = 0.444, *p* < 0.01) and negatively related to resilience (*r* = − 0.474, *p* < 0.01) Psychological resilience: perceived resilience (*F*(7, 490) = 23.60, *p* < 0.001)
Dawson und Thompson 2017, UK [42]	weak	NEO Five-Factor Inventory (NEO-FFI), MBI and Job Stress Survey (JSS)	A sample consisting of 363 veterinary (139 males and 220 females, 4 did not specify sex) in a cross-sectional study	H1: Veterinarians' experiences of occupational stress will be explained more through personality factors than environmental factors: The personality can explain 7.3% (R2 = 0.073) of the variance in occupational stress. The final model indicates that personality has a significant effect on occupational stress (F[1, 309] = 24.411, *p* < 0.001). The beta coefficient confirms that personality significantly predicts occupational stress (β = 0.271, t[309] = 4.941, *p* < 0.001) H2: The personality traits of neuroticism and conscientiousness will be more related to occupational stress than the traits of extraversion, openness, and agreeableness: Neuroticism can explain 7.3% (R2 = 0.073) of the variance in occupational stress. The final model indicates that neuroticism is a significant predictor of occupational stress (F[1, 309] = 24.411, *p* < 0.001). Likewise the beta coefficient confirms this (β = 0.271, t[309] = 4.941, *p* < 0.001). Environment can explain 2.2% of the variance in OS when neuroticism is removed (R2 = 0.022). The final model indicates that environment has a significant effect on occupational stress (F[1, 309] = 6.958, *p* = 0.009). The beta coefficient also shows that environment significantly predicts OS (β = 0.148, t[309] = 2.638, *p* = 0.009) A1: To establish the key facets within neuroticism and conscientiousness that most contribute to stress: 6.5% of the variance in occupational stress can be attributed to the personality component depression and another 2.4% to anger and hostility, two of the five facets of neuroticism. The final model shows that both depression and anger hostility have a significant effect on OS (F[2, 308] = 15.027, *p* < 0.001). Beta coefficients confirm that depression and anger hostility are significant predictors of OS, with depression presenting the strongest correlation (β = .183, t[308] = 3.054, *p* = 0.002) followed by anger hostility (β = 0.171, t[308] = 2.853, *p* = 0.005) A2: To explore demographic factors (such as years qualified and type of practice) as potential mediators and/or moderators of any relationships found: Years qualified (YO) and anger hostility were significant predictors of occupational stress (F[6, 302] = 7.531, *p* < 0.001). Beta coefficients confirm that years qualified is a significant mediator in the relationship between depression and occupational stress (β [YQ] = − 0.200, t[302] = − 3.684, *p* < 0.001; β [anger hostility] = 0.156, t[302] = 2.582, *p* = 0.010). Type of practice presented no significant effects on facets within neuroticism and conscientiousness YQ moderated the EE and DP relationships with respect to OS, resulting in weaker correlations (final model [YQ, EE]: F[2, 306] = 64.713, *p* < 0.001). The beta coefficients confirm these correlations (β [YQ] = − 0.154, t[306] = − 3.178, *p* = 0.002; β [EE] = 0.501, t[306] = 10.331, *p* < 0.001; final model [YQ, DP]: F[2, 306] = 41.339, *p* < 0.001) and the corresponding beta coefficients demonstrate this correlation between YQ and DP (β [YQ] = − 0.140, t[306] = − 2.700, *p* = 0.007; β [DP] = 0.410, t[306] = 7.901, *p* < 0.001). However, results show that there was a moderated relationship between PA and OS in relation to YQ, resulting in a stronger correlation (final model [YQ, PA]: F[2, 306] = 10.980, *p* < 0.001). The beta coefficients confirm this moderated relationship, revealing an increase in the strength of the correlation (β [YQ] = − 0.230, t[306] = − 4.173, *p* < 0.001; β [PA] = − 0.122, t[306] = − 2.206, *p* = 0.028)
Dow et al. 2019, Australia [38]	weak	Kessler Psychological Distress Scale (K10), Compassion Fatigue Short Scale (CFSS), and items designed by the researchers specifically for the study (personal grief when the life of a client's animal ends and their physical and mental wellbeing)	A sample consisting of 103 veterinary (63,1% female) in a cross-sectional study	Mental/physical health was affected by euthanasia (40.2% (strongly agree + agree)) CFSS: There was a statistically significant association between total score on the CFSS and hours worked when adjusting for age (global *p*-value < 0.0001). The most significant comparison was veterinarians who worked 10–20 h had a mean CFSS score 43 units less than veterinarians who worked 20–30 h (estimate = − 43, 95% CI: − 62, − 23, comparison *p*-value < 0.0001). The most significant comparison was veterinarians aged 18–34 had a mean CFSS score 32 units more than veterinarians aged > 64 (estimate = 32, 95% CI: 19, 44, comparison *p*-value < 0.0001) K10: There was a statistically significant association between psychological distress and age when marital status and animal type were considered (global *p*-value = 0.0029). The most significant comparison was that veterinarians aged 18–34 years had a mean K10 score 8 units higher than veterinarians aged > 64 years (estimate = 8, 95% CI: 4, 13, comparative *p*-value = 0.0005) There was a statistically significant association between psychological distress and marital status when age and animal type were considered (global *p*-value = 0.0314). Married and partnered veterinarians had a mean K10 score 3 units lower than non-married or partnered veterinarians (estimate = -3, 95% CI: -5, -0.3) There was a statistically significant association between psychological distress and animal type when age and marital status were considered (global *p*-value = 0.0218). Veterinarians dealing with companion animals, horses, and mixed animals had a mean K10 score 10 units higher than veterinarians dealing with other animals or involved in research (estimate = 10, 95% CI: 1, 18)
Fritschi et al. 2009, Australia [37]	weak	General Health Questionnaire (GHQ), Warr's work-related affect scales, self-reported questions	A sample consisting of 2125 veterinary (1217 male, 908 female) in a cross-sectional study	psychological health associated with demographic and practice factors: GHQ score > 2: Gender (*p* < 0,001): female 37.6%, male 29.7%; Practice type (*p* < 0,4): mixed 34.8%, small 33.1%, non-animal 32.1%, large 29.6% Mean Anxiety/Contentment score: Gender (*p* < 0,001): male 4.04%, female 3.72%; Practice type (*p* < 0,001): non-animal 4.11%, large 4.04%, mixed 3.92%, small 3.83% Mean Depression/Enthusiasm score: Gender (*p* < 0,001): male 4.46%, female 4.31%; Practice type (*p* < 0,007): non-animal 4.58%, large 4.47%, mixed 4.41%, small 4.34% GHQ Wald coefficient (95% CI): Gender: female 1.13 (0.89, 1.44), male 1.0; Practice type: non-animal 1.40 (0.77, 2.56), mixed 1.12 (0.87, 1.43), large 1.06, (0.78, 1.45), small 1.0; Working hours (per hour): 1.01 (1.00, 1.02) Anxiety/Contentment Beta (95% CI), adjusted R^2^ 0.479: Gender: male (Baseline), female − 0.12 (− 0.198, − 0.06); Practice type: non-animal 0.09 (− 0.07, 0.24), mixed 0.04 (− 0.02, 0.11), large 0.01 (− 0.07, 0.09), small (Baseline); working hours − 0.01 (− 0.01, − 0.00) Depression/Enthusiasm Beta (95% CI), adjusted R^2^ 0.566: Gender: female 0.01 (− 0.05, 0.07), male (Baseline); Practice type: non-animal 0.10 (− 0.04, 0.23), large − 0.03 (− 0.10, 0.04), mixed 0.01 (− 0.05, 0.06), small (Baseline); working hours − 0.00 (− 0.01, − 0.00)
Hansez et al. 2008, Belgium [21]	weak	Positive and Negative Occupational Stress Inventory (PNOSI), SWING, subscale of emotional exhaustion	A sample consisting of 216 veterinary (75,5% male, 24,5% female) in a cross-sectional study	job engagement: mean 54.06 (± 8.89); Level low 3.7%, Level medium 71.3%, Level high 24.2%; Type of activity: small animals 56.55 (± 8.95), mixed 53.41 (± 8.91), bovine 52.09 (± 8.49) job strain: mean 52.19 (± 8.15); Level low 5.6%, Level medium 79.2%, Level high 14.8%; Type of activity: mixed 54.24 (± 6.97), bovine 53.14 (± 8.20) small animals 50.64 (± 8.17) burnout: mean 22.22 (± 9.47); level low 31%, Level medium 51.9%, Level high 14.4%; Type of activity: bovine 24.14 (± 10), mixed 22.79 (± 9.09), small animals 20.93 (± 9.20)
Harling et al. 2007, Germany [43]	weak	Frequency-quantity index, CAGE-Test, Demoralization Scale, Psychosocial Stress Scale	A sample consisting of 1131 veterinary (male 47,5%, female 52,5%) in a cross-sectional study	Psychosocial stress: burdened (19.1%), mean: 1.4 more hours, more stress (*r* = 0.443, *p* < 0.001); stress for self-employed more than for employed veterinarians (*r* = -0.2, *p* < 0.001) mean demoralization scale: 1.2; employed more demoralized than self-employed (*r* = 0.119; *p* < 0.001); young vets more demoralized than older vets (*r* = -0.124; *p* < 0,001) severe psychosocial stress often associated with demoralization (*r* = 0.442; *p* < 0.001)
Hatch et al. 2011, Austria [44]	weak	K10, DASS, CBI	A sample consisting of 1947 veterinary (51.4% male, 48.6% female) in a cross-sectional study	K10 scores: all respondents (*n* = 1944): low (35.2%), moderate (42%), high (14%), very high (5%) DASS-depression scores: all respondents (*n* = 1942): normal (74.5%), mild (7.9%), moderate (10%), severe (3.8%), extremely severe (3.9%) DASS-anxiety scores: all respondents (*n* = 1942): normal (83.3%), mild (4.6%), moderate (7.8%), severe (2.2%), extremely severe (2.1%) DASS- stress: all respondents (*n* = 1942): normal (68.2%), mild (11.5%), moderate (10.5%), severe (6.8%), extremely severe (2.4%) Burnout CBI: reference data: personal (22.2%, *n* = 1945), work (19.7%, *n* = 1946), client (16.6%, *n* = 1933) all respondents: personal (37%), work (35.6%), client (24.8%) Logistic Regression more likely highest categories (DASS depression): capital cities, rural cities/town high/very high K10 scores (> 22): Female (OR = 1.6, 95%CI:1.2–2.0) and veterinarians < 10 years high/very high personal burnout scores: Female (OR = 2.3, 95%CI: 1.9–2.9), capital city (OR = 2.55, 95%CI: 1.1–6.1), rural city/town (OR = 1.4, 95%CI: 1.0–1.9) Lower work and client burnout scores (OR < 1): all types of practice/ work other than companion animal practice
Kassem et al. 2019, USA [45]	weak	Kessler psychological distress scale	A sample consisting of 9522 veterinary (30.8% male, 69.2% female) in a cross-sectional study	negative attitude toward treatment effectiveness: male vs. female OR = 1.79, solo vs. nonsolo OR = 1.60, with vs. without psychological distress OR = 2.11, suicide ideation vs non OR = 1.83 Negative attitude toward social support: males vs females OR = 0.72, solo vs nonsolo OR = 1.23, not belong vs belong veterinary association OR = 1.29, psychological distress vs none OR = 1.55; suicidal ideation vs none OR = 1.55, age 40–59 vs 20–39 = OR = 1.18. small animal practice associated with neg. attitude toward treatment
Mair et al. 2021, UK [46]	weak	WEMWBS (pre and during covid pandemic)	A sample consisting of 451 veterinary (38.4% males, 61.0% females, 0.6% n.r.) in a cross-sectional study	WEMWBS mean: current survey (during pandemic): 47.17; 2019 survey (pre pandemic): 48.08 cheerful: none of time (0–2%), rarely (2–17%), some of the time (17–60%), often (60–93%), all of the time (93–100%) interested in new things: none of time (0–8%), rarely (8–27%), some of the time (27–57%), often (57–88%), all of the time (88–100%) feeling loved: none of time (0–3%), rarely (3–11%), some of the time (11–48%), often (48–72%), all of the time (72–100%) able to make up my mind about things: none of time (0–1%), rarely (1–9%), some of the time (9–48%), often (48–79%), all of the time (79–100%) confident: none of time (0–2%), rarely (2–21%), some of the time (21–55%), often (55–88%), all of the time (88–100%) close to others: none of time (0–3%), rarely (3–27%), some of the time (27–60%), often (60–90%), all of the time (90–100%) feeling good about myself: none of time (0–5%), rarely (5–19%), some of the time (19–63%), often (63–91%), all of the time (91–100%) thinking clearly: none of time (0–1%), rarely (1–6%), some of the time (6–35%), often (35–82%), all of the time (82–100%) spare energy: none of time (0–7%), rarely (7–46%), some of the time (46–72%), often (72–93%), all of the time (93–100%) dealing with problems well: none of time (0–1%), rarely (1–7%), some of the time (7–45%), often (45–87%), all of the time (87–100%) interested in others: none of time (0–2%), rarely (2–9%), some of the time (9–46%), often (46–86%), all of the time (86–100%) relaxed: none of time (0–10%), rarely (10–41%), some of the time (41–77%), often (77–95%), all of the time (95–100%) being useful: none of time (0–4%), rarely (4–11%), some of the time (11–36%), often (36–69%), all of the time (69–100%) optimistic about future: none of time (0–4%), rarely (4–21%), some of the time (21–62%), often (62–91%), all of the time (91–100%)
Mastenbroek et al. 2014, Netherlands [20]	weak	Interviews and questionnaire: The Questionnaire Experience and Evaluation of Work (QEEW), Proactive Personality Scale, Groningen Reflection Ability Scale, nine-item version of the Utrecht Work Engagement Scale (UWES), exhaustion dutch version of MBI	A sample consisting of 860 veterinary (27% males, 73% females) in a cross-sectional study	correlations: workload: work-self conflict 0.463**, physical demands: work-self conflict 0.364**, feedback from work: decision latitude 0.327**, support form colleagues: feedback from work 0.394**, exhaustion: workload .376**, exhaustion: physical demands 0.338**, exhaustion: work-self conflict: 0.557**, exhaustion: decision latitude -0.416**, exhaustion: self-efficacy -0.313** (only over 0.300 and not all)
Nett et al. 2015, USA (Puerto Rico) [47]	weak	Kessler-6 psychological distress scale, history of depression and mental health treatment, attitudes toward mental illness and mental health treatment, stressors related to veterinary practice, and satisfaction related to practicing veterinary medicine	A sample consisting of 11.627 veterinary (male 31%) in a cross-sectional study	9% respondents with current serious psychological distress. Since leaving veterinary school, 31% respondents experienced depressive episodes, 17% experienced suicidal ideation, and 1% attempted suicide. Currently, 19% respondents were receiving treatment for a mental health condition. 32% respondents somewhat or strongly agreed that people are sympathetic toward persons with mental illness Reported psychological distress (score ≥ 13): female > male in all categories, previous depressive episodes (31%) > suicidal ideation (17%) > attempted suicide (1%). Among those who had attempted suicide, the median number of attempts was 1.0. Currently receiving treatment: *n* = 2228 (19%)
Perret et al. 2020, Canada [48]	weak	Davidson Resilience Scale, Perceived Stress Scale, HADS, MBI, ProQOL	A sample consisting of 1.130 veterinary (male 21.6%, female 78.4%) in a cross-sectional study	Subjective general health (excellent vs. reference person, poor; β = 18.28 [95% CI, 11.89 to 24.67]; t = 5.61; *p* < 0.001); Satisfaction with support from friends (very satisfied vs. reference person, not at all satisfied; β = 8. 87 [95% CI, 1.81 to 15.92]; t = 2.47; *p* = 0.014); Satisfaction with relationship or partner support (very satisfied vs reference person, not at all satisfied; β = 6.21 [95% CI, 0.60 to 11.82]; t = 2.17; *p* < 0.030) had strong positive associations with resilience; 2 children (2 vs reference, none; β = 2.74 [95% CI, 0.58 to 4.90]; t = 2.49; *p* = 0.013); 3 children (3 vs reference, none; β = 3.09 [95% CI, 0.23 to 5.95]; t = 2. 12; *p* = 0.034) or having a scheduled call (vs no call; β = 1.91 [95% CI, 0.13 to 3.70]; t = 2.11; *p* = 0.035) was also positively associated with resilience; self-reported presence of current mental illness had the strongest negative association with resilience (vs no current illness; β = -5.03 [95% CI, -7.37 to -2.69]; t = -4.23; *p* < 0.001); being married was negatively associated with resilience (married vs referent, single; β = -5.85 [95% CI, -10.88 to -0.81]; t = -2.28; *p* = 0.023) or practicing small animal medicine (small animal only vs referent, mixed; β = -2.53 [95% CI, -4.97 to -0.089]; t = -2.03; *p* = 0.042) association between mental health outcome scores (as dependent variable) and the CD-RISC scores: CD-RISC: mean 69.9 (range 20–99); PSS: mean 17.0 (range 0–7); HADS: mean 13.2 (range 0–39); MBI: emotional exhaustion: mean 26.1 (range 0–54), Depersonalization: mean 8.9 (range 0–8); Personal accomplishment: mean 36.6 (range 10–48); ProQOL: Burnout: mean 25.2 (range 10–45), secondary traumatic stress: mean 23.6 (range 10–46), Compassion satisfaction: mean 37.8 (range 14–50)
Shirangi et al. 2013 [49]	weak	Affective Well-Being Scale, PANAS, GHQ and CGHQ	A sample consisting of 1017 female veterinary in a cross-sectional study	GHQ: > 2: 37%; CGHQ: > 4: 63% Mean score on the Anxiety-Contentment Axis: 3.72 (± 0.8); Mean score on the Depression-Enthusiasm Axis: 4.31 (± 0.82) PANAS: PA mean score 33.5 (± 6.25); NA mean score: 18.7 (± 6.12)
Reijula et al. 2003, Finland [36]	weak	MBI, self-reported health, self-reported diseases	A sample consisting of 785 veterinary (male 225, female 550) in a cross-sectional study	severe burnout (age groups/year): 25–34: men (2.1) vs women (0.0); 35–44: men (2.0) vs women (0.0), 45–54: men (0.0) vs women (3.2), 55.65: men (0.0) vs women (3.1), total: women (1.8) vs men (1.7) self-reported health: men: 55–65 years: rather good (46.8%) > average (40.4%) > good (10.6%) > poor (2.1%) 45–54 years: rather good (47.6%) > average (32.1%) > good (14.3%) > rather poor (6%) 35–44 years: rather good (38.6%) > average (29.8%) > good (28.1%) > rather poor (3.5%) 25–34 years: rather good (40.0%) > average (30.0%) > good (26.7%) > rather poor (3.3%) women: 55–65 years: average (53.3%) > rather good (26.7%) > good (13.3%) > rather poor (6.7%) 45–54 years: rather good (36.9%) > average (31.0%) > good (22.6%) > rather poor (7.1%) > poor (2.4%) 35–44 years: rather good (39.6%) > good (31.0%) > average (25.4%) > rather poor (2.5%) > poor (1.5%) 25–34 years: rather good (40.8%) > good (36.1%) > average (21.0%) > rather poor (2.1%) self-reported diseases: mental disorder: women (8%), men (7%)
Rivera et al. 2021, USA [50]	weak	PHQ-8	A sample consisting of 101 veterinary (40.6% male, 59.4% female) in a longitudinal cohort study (2001, 2004, 2007, and 2011)	Mental health problem: No (84.2%. *n* = 85), Yes (15.8%, *n* = 16), *p* = 0.026 Suicidal ideation: Not at all (91.8%, *n* = 67), several days or more (8.2%, *n* = 6), *p* = 0.282 Lack of social support: No (not bothered) (63.4%, *n* = 64), Yes (bothered) (36.6%, *n* = 37), *p* = 0.023
Schwerdtfeger et al. 2020b [13]	weak	PHQ-9, SBQ-9	A sample consisting of 3.118 veterinary (20.5% male, 79.5% female) in a cross-sectional study	PHQ-9: 27.78% were screened positive for depression (17.45% displayed moderate symptoms of depression, 10.33% indicated moderately severe to severe symptoms of depression). Compared with the general population: OR = 0.349; 95% CI 0.309 to 0.940 PHQ-9 (item 9): 19.2% having suicidal ideation in the past two weeks (15.91% reporting to have had such feelings on several days during the last two weeks, 2.31% on nearly half of the days and 0.96% nearly every day during the last two weeks). Compared with the general population: OR = 0,497; 95% CI 0,445 to 0,554 SBQ-9: 32.11% were classified as having an increased suicide risk (compared with the general population: OR = 0,150; 95% CI 0,123 to 0,183). 38.3% have never thought about, planned or attempted suicide. 24.2% report that they have planned to kill themselves at least once. 2.7% stated that they have attempted to kill themselves at least once in the past
Schwerdtfeger et al. 2020a, Germany [51]	weak	COPSOQ, PHQ-9, SBQ-R	A sample consisting of 3179 veterinary (22.2% male, 78.8% female) in a cross-sectional study	stress makes it difficult to meet personal/family obligations = female: agree (32%), disagree (21%), partly (19%), fully agree (18%), totally disagree (9%) male: agree (29%), disagree (25%), partly (17%), totally disagree (15%), fully agree (14%) frequency feeling emotionally exhausted = female: often (36%), sometimes (33%), rarely (21%), always (5%), never (4%) male: rarely (33%), sometimes (29%), often (25%), never (11%), always (3%) current suicidal thoughts (19.2%, *n* = 598), increased suicide risk (32.1%, *n* = 1001), clinically relevant depression symptoms (27.8%, *n* = 886)
Witte et al. 2020, England & USA [52]	weak	Kessler 6 psychological distress scale and self-formulated question (revalence of serious psychological distress, a history of depressive episodes, a history of suicidal ideation, and a history of attempted suicide and negative mental health outcomes and work- and school-related emotional outcomes for respondents)	A sample consisting of 440 veterinary (Cis female: 62.0%, Cis male: 30.7%, Transgender (male to female): 0.5%, Transgender (female to male): 1.6%, Do not identify as male or female: 4.5%, Prefer not to answer: 0.7% in a cross-sectional study	Highest correlation between the scores for emotional exhaustion and job satisfaction (-0.66) Prevalence of serious psychological distress (Kessler 6 score ≥ 13) in different to the prevalences of the veterinarians in general (Nett et al. 2015 [47]): Transgender and nonbinary individuals (41%, *p* < 0.01), Nonheterosexual cis women 16%, *p* = 0.005), Transgender and nonbinary individuals (50%, *p* = 0.001) Prevalence of depressive episodes in different to the prevalences of the veterinarians in general (Nett et al. 2015 [47]): Nonheterosexual cis women (45%, *p* = 0.003)
**Study without valid survey instruments**				
Batchelor und McKeegan 2012, UK [12]	weak	Ethical dilemmas: the frequency with which they faced ethical dilemmas in an average week (0, 1 to 2, 3 to 5, 6 to 10, > 10) & three common scenarios: (1) convenience euthanasia of a healthy animal, (2) financial limitations of the client restricting the treatment options and (3) the client wishing to continue treatment despite compromised animal welfare/quality of life (scale 0–10, 0 not at all stressful, 10 extremely stressful)	A sample consisting of 58 practicing veterinary surgeons (15 male, 43 female) in a cross-sectional study	The median stress ratings (0, 1 to 2, 3 to 5, 6 to 10, > 10): healthy animal euthanasia (female 8, male 7), financial limitations (7 female, 7 male) and client wishing to continue treatment (9 female, 8 male) Most commonly encountered dilemma: financial limitations (55%), healthy animal euthanasia (7%), client wishing to continue treatment (14%), other (5%), none give (19%)
Epp und Waldner 2012, Canada [32]	weak	Not standardized, Scale 1–5, 1 no stress, 3 moderate, 5 severe stress	A sample consisting of 823 veterinary (44.7% male, 54.8% female, without 4) in a cross-sectional study (75.9% practice, 11.1% academia, 5.2% industry, 7.8% government)	2% reported no job-related stress, 5% reported severe stress, whereas the majority (53%) reported moderate stress. No significance of median stress scores among veterinarians working in practice, industry, government, or academia (*p* = 0.74). For each group, the median reported stress score was 3 on a scale of 5. Stress was higher among those who had graduated in the past 2 decades compared with those who graduated before 1990 (*p* = 0.005), among women compared with men (*p* < 0.001), and among those who worked more than 40 h per week (*p* = 0.001). The types of stress reported by respondents differed by work environment; workload and client-related problems were most common among veterinarians working in a practice Workload-related (Yes), *p* = 0,001: practice (79%), academia (64%), industry (70%), government (63%) Client-related (Yes), *p* < 0,001: practice (62%), academia (30%), industry (26%), government (27%)
Hagen et al. 2020 [53], UK	weak	Questionnaire with closed and open questions within three sections: ‘current employment’, ‘about you’ and ‘you as an employer’	A sample consisting of 2472 veterinary (22,9% male, 76,8% female) in a cross-sectional study	reasons to stay in a position (*n* = 701): team 56.7%, location 48.3%, family 34.4%; reasons to leave a position (*n* = 536): work-life balance 41.2%, management 39.6%, salary 33.8%; Assumptions by employers about leaving (not only veterinarians): family 32.6%, asked them to leave 24.1%, location 22.2%, work-life balance 22%, other 22%; most disliked aspects about profession (*n* = 2365): dealing with people 50.4%, work-life balance 26.6%, physical/mental stress 19.6%; what they would change (*n* = 2169): working hours 29.6%, more team support 16.9%, management 14.7%
Heath 2008, Australia [31]	weak	Not standardized (Respondents were asked to indicate whether they strongly agreed (SA: score = 1) agreed (A: 2), were neutral (N: 3), disagreed (D: 4) or strongly disagreed (SD: 5) with each statement)	A sample consisting of 350 veterinary (25% males, 75% females) in a cross-sectional study	I felt significant and regular stress: 29 (SA), 41 (A), 14 (N), 14 (D), 2 (SD) stress: significant and regular stress: 75% female, 57% male (*p* < 0.01); main factors (*p* < 0.001): help and support from boss, work-life-balance, adequacy of compensation; stress—boss as role model for behaviour (*p* < 0.05); stress—type/size of the practice; hours worked (no significance); hours worked—(troublesome) work-life-balance (*p* < 0.001)
Kogan et al. 2018, USA [33]	weak	Not standardized (involvement with near misses (NM) and adverse events (AE))	A sample consisting of 606 veterinary (22.6% male, 77.4% female) in a cross-sectional study	66.4% with near misses (NM), 29.5% with adverse events (AE) in the past 12 month. NM: 68.0% with short-term (≤ 1 week after the incident) negative impact; 36.4% with long-term (> 1 week after the incident) negative impact on personal life For AE: 84.1% short-term and 56.2% long-term. NM: 37.6% less confidence in their ability as a doctor, 31.5% felt their confidence in their abilities had suffered, 29.5% ag less satisfied with their job, 26.5% felt burned out. AE: 44.3%) less confident in their ability as a doctor, 44.3% felt their confidence in their abilities had suffered, 42.4 less satisfied with their job, 37.7% felt burned out, 36.9% decrease in overall happiness, 35.1% felt that their professional reputation had been negatively impacted, 33.7% had problems sleeping, and 33.5% felt persistently guilty. – > 70.3% stress level outside of work had not impacted the number of NMs or AEs. 4.0% high stress outside of work had markedly increased the frequency of these incidents, 24.0% slightly increased the frequency of these incidents
Morello et al. 2019, USA [34]	weak	Not standardized (reciprocal effects of career, family and gender on elements of their professional life (diploma, income, inequality etc.)	A sample consisting of 836 veterinary (59% males, 41% females) in a cross-sectional study	income: private practice > academia***, small animal > large animal***, males > females***; practice ownership: males > females***, working time: private practice owner > other**, comments about their gender related to performance: females > males***. passion for the job the most importance factor, also finicial compensation and locaton. Emergency duties were the least influential factor. Women were more likely to report negative underemployment (i.e. the desire to work fewer hours) than men
Moses et al. 2018, North America (USA—Canada) [35]	weak	Not standardized (ethical conflict and moral distress)	A sample consisting of 889 veterinary in a cross-sectional study	Moral distress levels and coping methods: not being able to do the right thing: severe stress (73%), moderate—severe stress (78%), not being able to provide care they thought was appropriate: moderate—severe distress (69%), distressed or anxious about work: often (43%) > some-times (34%)

Methodologically, a total of 28 [54–81] different valid collection instruments with reference to workload, psychosocial stressors, mental wellbeing, burnout, psychological problems, anxiety, depression, and suicidal factors were used (Table 2). All survey instruments, of the studies listed in this section, are presented including description and abbreviation in Table 2.

**Table 2:** List of valid survey instruments used with indication of cut-off values

Valid survey instruments (with reference to workload, psychosocial stressors, mental well-being, burnout, psychological problems, anxiety, depression, and suicidal factors)	Cut-off values
Hospital anxiety and depression scale (HADS) [54]	caseness: ≥ 8; possible case: 8–10; probable case: ≥ 11
Warwick-Edinburgh mental well-being scale (WEMWBS) [55]	14 individual item scores from 1 (none of the time) to 5 (all of the time) (scores 14 to 70): The higher the values in the score, the more pronounced the mental well-being
Health and Safety Executive management standards indicator tool (HSE MSIT) [56]	35 questions grouped into seven key stressor domains: demands (8 items), control (6 items), managerial support (5 items), peer support (4 items), relationships (4 items), role (5 items), and change (3 items), which have the potential to have a negative impact on employee mental health and well-being. Each question scores 1–5 from the least favourable working conditions (high risk of stress at work) to the most favourable working conditions (low risk of stress at work), respectively. The overall score for each of the seven stressor domain scales is calculated for each respondent by adding the item scores for each question answered in that scale and dividing by the total number of questions answered in that scale
Survey Work-home Interaction Nijmegen (SWING) [57]	A total of 22 items in 4 subscales. An aggregate result is calculeated based on the total score obtained in eaach of the four subscales
Maslach Burnout Inventory (MBI) & MBI-Human Services Scale (MBI-HSS) (designed for professionals in the human services) [58]	Occupational exhaustion (EE): < 17 (low degree), 18 – 29 (moderate degree), > 30 (high degree) Depersonalisation (DP): < 5 (low degree), 6 – 11 (moderate degree), > 12 (high degree) Personal accomplishment assessment (PA): < 33 (low degree), 34 – 39 (moderate degree), > 40 (high degree)
Copenhagen Burnout Inventory (CBI) [59]	Five point Likert scale with three subscales: personal (six items), work burnout (seven items), and client burnout (six items). Scores ranged from 1 – 100 (high score = burnout risk)
Professional Quality of Life (ProQOL) [60]	3 subscales: Compassion Satisfaction (pleasure you derive from being able to do your work well), Burnout (exhaustion, frustration, anger and depression related to work): Secondary Traumatic Stress (feeling fear in relation to work‐related primary or secondary trauma) For each of the sub-scales scores are categorised as Low (22 or less), Moderate (between 23 and 41) or High (42 or more)
Connor-Davidson Resilience Scale (CD-RISC) [61]	25 items, each rated on a 5-point scale (0–4), with higher scores reflecting greater resilience
Swedish Demand-Control-Support Questionnaire (DCSQ) [62]	3 subscales (psychological demands, decision latitude, social support) with 17 items High scores: high occupational stress, high work control and high social support
Depression, Anxiety, Stress Scale (DASS-21) [63]	21 items in three self-report scales Depression (score): normal (0–9), mild (10–13), moderate (14–20), severe (21–27), extremly severe (28 +) Anxiety (score): normal (0–7), mild (8–9), moderate (10–14), severe (15–19), extremly severe (20 +) Stress (score): normal (0–14), mild (15–18), moderate (19–25), severe (26–33), extremly severe (34 +)
Positive and Negative Affect Scale (PANAS) [64]	2 scales (positve affect, negative affect) with each 10 items. Scores can range from 10 – 50, with higher scores representing higher levels of positive or negative affect
Frost Multidimensional Perfectionism Scale (FMPS-Reduced) [65]	35 items in four subscales for perfectionism (concern over mistakes and doubts about actions, excessive concern with parents’ expectations and evaluation, excessively high personal standards, concern with precision, order and organisation): Higher percentiles indicate more problems while a percentile closer to 50 represents average (and healthy) responses. Percentile scores above the 90th percentile are of clinical significance and represent dysfunctional perfectionism
Kessler Psychological Distress Scale (K10) [66]	Score (10–50); < 20: well; 20–24: mild mental disorder; 25–29: moderate mental disorder; ≥ 30: severe mental disorder
Compassion Fatigue Short Scale (CFSS) [67]	Score (13–130) from low/no compassion fatigue to frequent symptoms of compassion fatigue: very low = < 27, low = 27–30, mild = 31–35, high = 36–40 and > 40 = very high
General Health Questionnaire (GHQ-12) [68] & Chronicity and the General Health Questionnaire (CGHQ) [69]	2 items, each assessing the severity of a mental problem over the past few weeks using a 4-point scale (from 0 to 3). Psychological distress was defined as scoring above 2 when the responses are summed across the 12 items
Patient Health Questionnaire depression scale (PHQ-8) [70] & Patient Health Questionnaire depression scale (PHQ-9) [71]	8 item scala with a score from 0 – 24 (≥ 10 Depression) & 9 item scala with a score from 0 – 24 (≥ 10 Depression) and one additional item to assess suicidal ideation (Item 9)
Positive and Negative Occupational Stress Inventory (PNOSI) [72]	19 items (8 items assessed job engagement, 11 items assessed job strain) Moderate level of job strain/job engagement (values 40 – 60), very low job engagement (< 40)
Suicide Behaviours Questionnaire-Revised (SBQ-R) [73]	Scala with 4 items. The total score of the four items ranges from 3 to 18, with a score of 8 and above used to identify patients with increased suicide risk
Perceived Stress Scale (PSS) [74]	10 items (5-point Likert): 0–13 (low stress); 14–26 (moderate stress) 27–40 (high perceived stress)
Copenhagen Psychosocial Questionnaire (COPSOQ) [75]	A long version with 141 items forming 30 scales, the so-called “research questionnaire”. A medium-length version with 95 items on 26 scales, the “questionnaire for work environment professionals”. A short version with only 44 items and 8 scales "questionnaire for workplaces
Job Stress Survey (JSS) [76]	10-item subscales (0 to 9 + days)
NEO Five-Factor Inventory (NEO-FFI) [77]	The sum of the items of the 5-point scale results in a category for the degree of expression of the characteristic in the participant: very low, low, average, high or very high
Job-Related Affective Well-Being Scale [78]	A mean score for each scale is found by reverse scoring each of the negative adjectives, adding each response, and dividing by the number of responses. Higher scores on each scale indicates higher affective well-being in that category
Utrecht Work Engagement Scale (UWES) [79]	In order to interpret the scores of a particular group of employees on (a dimension of) the UWES, the mean score from the database can be used

The prevalence of anxiety and depression among United Kingdom (UK) veterinarians (*N* = 1,796) reported by Bartram et al. [22] was 26.3% and 5.8%, respectively, with a HADS subscale score of ≥ 11 (probable case). Best et al. [39] also used the HADS score to determine the mental health of Canadian veterinarians (*N* = 412) on anxiety and depression; they found that 32% of participants could be classified as likely anxious based on a HADS-A score ≥ 11, and 9% of participants were classified as likely depressed based on a HADS-D score of ≥ 11. The mean value of the HADS-A was slightly higher at 8.8 points compared to 4.6 points in the Bartram et al. [22] sample. The same is true for the mean value of the HADS-D score, which was 5.2 points in the study sample of Best et al. compared to 4.6 points in Bartram and colleagues.

Based on the MBI, Human Services Survey decision criteria (a “high” score on emotional exhaustion plus either a “high” score on depersonalization or a “low” score on personal realization), 36.9% of participants could be classified as affected by burnout in Best et al. [39] (female participants at 37.8% tended to be higher than male participants at 32.7%). The prevalence of work-related burnout (using the MBI) among Finnish veterinarians [36] found that 40% had moderate sympto ms and 1.7% had severe symptoms. The veterinarians in private practice were the least affected by burnout (2.4% severe and 30.5% moderate), as were community veterinarians (1.3% severe and 37% moderate). Ten percent of veterinarians suffered from work-related fatigue or emotional exhaustion, and 42% suffer from moderate emotional exhaustion. The prevalence of work-related burnout wasmost common among veterinarians working with small animals, equine veterinarians, clinical veterinarians, and veterinarians in private practice. Seven percent of respondents reported severe symptoms of cynicism, and 26% reported moderate cynicism. In self-reported work-related risks, 8% of women and 7% of men reported mental disorders. When asked about their current stress level, 73% of veterinarians (71% of women and 77% of men) reported being rather or more stressed.

In self-reported health information in a study of occupational stress and risk factors among Turkish veterinarians (*N* = 223) by Cevizci et al. [40], 19.7% reported suffering from stress, 15.4% reported shortness of breath, 12.8% reported depression, 12% reported burnout, 10.3% reported unhappiness/restlessness, 7.7% reported chronic fatigue/insomnia, and 0.9% reported attention deficit. In addition to nonstandardized data collection instruments, Cevizci et al. examined the sample using the Turkish version of the Swedish Demand-Control-Support Questionnaire but compared veterinarians in the public sector and veterinarians in the private sector. The mean scores collected between the two groups were similar, but only decision latitude was statistically significantly higher among veterinarians in the public sector (3.61 vs. 2.94, *p* = 0.008).

Crane et al. [41] examined morality-related stressors (e.g., suspicion of patient/animal abuse, clients unable to pay for recommended treatment, or performing euthanasia) with psychological distress and resilience among Australian veterinarians (*N* = 540). In addition, the role of perfectionism in strengthening the association between exposure to morally significant stressors and psychological distress was examined. Crane et al. found that moral significance of stressors was statistically significantly related to psychological resilience. Higher levels of perfectionism were statistically significant associated with a tendency to view stressors as more morally statistically significant. They also examined the role of perfectionism in strengthening the association between exposure to morally statistically significant stressors and psychological distress. Perfectionism was positively related to stress (*r* = 0.509, *p* < 0.01), anxiety (*r* = 0.473, *p* < 0.01), and negative affect (*r* = 0.444, *p* < 0.01), and negatively related to resilience (*r* =  − 0.474, *p* < 0.01).

The relationship between demographic, occupational, and lifestyle factors and resilience, as well as the relationship between resilience and mental health, was examined by Perret et al. [48] in Canadian veterinarians (*N* = 1.130). Here, subscale scores (PSS, HADS, MBI, and ProQOL) were each treated as outcomes in univariable linear regression analyses, using the CD-RISC score as the independent variable. Veterinarians’ assessed general health, satisfaction with support from friends, and satisfaction with support from relationships or partners had strong positive associations with resilience. In addition, there is a strong negative association between mental illness and the CD-RISC score. The CD-RISC score was negatively related to scores for perceived stress, anxiety, depression, burnout, and secondary traumatic stress. In addition, there was a statistically significant relationship between the mental health scores of the PSS (mean 17.0, *p* < 0.001), HADS (mean 13.2, *p* < 0.001), MBI (emotional exhaustion mean 26.1, *p* < 0.001, depersonalization mean 8.9, *p* < 0.001, personal coping mean 36.6, *p* < 0.001) and CD-RISC (mean 69.9). The mean scores of the ProQOL were 25.2 for burnout, 23.6 for secondary traumatic stress, and 37.8 for compassion satisfaction; however, there were no gender-specific data. Best et al. [39] also used the ProQOL to assess occupational quality of life there were higher scores for burnout (44.8% vs. 38.9%) and secondary traumatic stress (72.9% vs. 60.7%) in female veterinarians. Although male and female participants had similar scores for compassion satisfaction, men were more likely to score in the “high” category on this subscale (36.3% vs. 31.4%).

Previous studies focused on environmental factors in isolation, overlooking the influence of personality. Dawson et al. [42] wanted to investigate whether personality is a better predictor of occupational stress than environment. For this they used the NEO Five-Factor Inventory, the MBI, and the Job Stress Survey and found that personality was a better predictor of job stress than environment in British veterinarians (*N* = 311). Neuroticism is the trait that statistically significant predicts job stress (*p* < 0.001). Dawson et al. (2017) were found that depression and anger hostility are the components of neuroticism that contribute most to stress. In addition, demographic factors were examined, which are considered as potential mediators and/or moderators of any relationships found. Demographic factors (such as years qualified and type of practice) mediated the relationship between depression and occupational stress (*p* < 0.001) and moderated the relationship between personal achievement and occupational stress (*p* = 0.028). Further, the results of Dawson et al. (2017) indicate that newly qualified veterinarians are at greater risk of suffering from high levels of occupational stress than those well established in the profession.

Dow et al. [38] examined the impact of veterinarians’ (*N* = 103) psychological wellbeing when dealing with grieving clients using the concept of compassion fatigue (Compassion Fatigue Short Scale (CFSS)). The results of the CFSS show a statistically significant relationship between the total score on the CFSS and hours worked after adjusting for age. Veterinarians who worked 10–20 h per week had a 43-unit lower mean CFSS score than veterinarians who worked 20–30 h per week. Veterinarians aged 18–34 years had a 32-unit higher mean CFSS score than veterinarians aged > 64 years. Regarding the K10 scale, a statistically significant relationship was found between psychological distress and age when adjusting for marital status and animal type (practice type). Younger veterinarians aged 18–34 years had a mean K10 score 8 units higher than older veterinarians aged > 64 years. There was a statistically significant association between psychological distress and marital status when age and animal practice type were considered (*p* < 0.01). Married and partnered veterinarians had a mean K10 score 3 units lower than veterinarians who were not married or partnered. There was a statistically significant association between psychological distress and animal type when age and marital status were considered (*p* = 0.031). Veterinarians who dealt with pets, horses, and mixed animals had a mean K10 score 10 units higher than veterinarians who dealt with other animals or were involved in research. Forty percent (40.2%) of respondents reported that their mental/physical health had been affected by euthanasia and 33.69% had experienced difficulty in performing euthanasia because of personal distress. Almost eighty-eight percent (87.6%) of the veterinarians surveyed had experienced grief at the end of an animal’s life.

Hatch et al. [44] also used the K10 scale, among other measures, in their study to determine the prevalence of depression, anxiety, stress, and burnout and their association with demographic characteristics of Australian veterinarians (*N* = 1,947). The K10 results were in the low (35.2%), medium (42%), high (14%), and very high (5%) ranges of psychological distress. According to the CBI of veterinarians, 22.2% reported personal burnout, 19.7% reported work-related burnout, and 16.6% reported client-related burnout. Veterinarians from large cities (OR = 2.6, *p* = 0.03) and from rural cities (OR = 3.1, *p* = 0.01) were statistically significant more likely to fall into the highest categories of depression scores than veterinarians from rural areas or farms (reference category). Veterinarians’ depression scores (DASS depression score) were distributed as follows: normal (74.5%), mild (7.9%), moderate (10%), severe (3.8%), and extremely severe (3.9%). A total of 83.3% of the veterinarians were classified as anxious (DASS anxiety score). Stress scores among veterinarians were as follows: 68.2% normal and 11.5% mild.

Fritschi et al. [37] used the GHQ, Warr’s work-related affect scales, and self-report questions to identify levels of stress, anxiety, and depression in veterinarians (*N* = 2,125). Chi-squared tests were used to determine statistical significance of any differences. The results of the GHQ indicated statistically significant higher psychological distress in women than in men (37.6% vs. 29.7%). Within the gender distributions, the mean value for anxiety (3.72 women vs. 4.04 men) and depression (4.31 vs. 4.46) were proportionally similar. The results from the linear regression analysis of the Warr scales were statistically significant worse for the psychological control variables and the other variables (social support, positive and negative affect) on the anxiety/satisfaction scale for women. Anxiety and depression tended to increase with longer working hours (*p* < 0.001).

As the only study without gender differentiation, Shirangi et al. [49] used established psychological scales to measure levels of distress and work-related stress (anxiety and depression) and the demographic and work characteristics of female veterinarians in relation to anxiety, depression and mental health. Thirty-seven percent of female veterinarians scored > 2 on the GHQ, indicating that they suffered from mild mental distress. Sixty-three percent of the female veterinarians scored above the cutoff value of 4 on the CGHQ. The mean score on the anxiety-satisfaction axis was 3.72, and for the depression-enthusiasm axis, it was 4.31 (± 0.82). The means for the positive and negative scales were 33.5 for PA and 18.7 for NA, respectively. The GHQ scores, which assessed psychological distress, indicated that the number of hours worked was related to the work stress felt by the female veterinarians. Women with 2 or 3 children had less anxiety and depression than those without children.

In their study, Hansez et al. [21] analyzed job engagement, job strain, burnout, work-home interference (WHI), and workplace stressors among veterinarians (*N* = 216). The mean score of the wellbeing variables studied was 54.06 points for job engagement, 52. Nineteen points indicated work stress, and 22.22 points indicated burnout. The average weekly working time of the respondents was 54.27 h. Men worked more hours than women (58.21 vs. 42.53 h/week). The mean professional commitment (surveyed by the PNOSI) of veterinarians was 54.06 points, with bovine veterinarians showing lower professional commitment than small animal veterinarians. Small animal veterinarians showed lower occupational stress than mixed veterinarians. The WHI is influenced by his subscales, the negative or positive load reactions. The results for work-life interference revealed statistically significant differences in the WHI subscales (*p* < 0.001).

The results of Harling et al. [43] showed that German veterinarians (*N* = 1.131) reported dealing with difficult clients, time pressure, frequent overtime, on-call duty, and weekend service as major reasons for stress. With sum scores in the upper half of the psychosocial stress scale, 19.1% of veterinarians were considered stressed. The more hours worked per week, the more stressed veterinarians were. Self-employed veterinarians experience stress more frequently than veterinarians who are employees. The values in the upper half of the demoralization scale reached 12.2%. Employed veterinarians are more demoralized than self-employed veterinarians, and young veterinarians (without exact definition to “young veterinarians”) are more demoralized than older veterinarians. In addition, severe psychosocial stress (based on a self-constructed scale following the model of occupational gratification crises by Siegrist) is often associated with demoralization.

Kassem et al. [45] examined the connection between demographic, occupational, and psychological characteristics and negative attitudes toward mental disorder among veterinarians (*N* = 9,522). The likelihood of having negative attitudes toward treatment efficacy was statistically significant (*p* < 0.05) higher for men than for women (OR = 1.79); for veterinarians practicing alone than for veterinarians not practicing alone (OR = 1.60); for those with (compared with those without) evidence of severe psychological distress (OR = 2.11); and for those who reported suicidal ideation after graduation from veterinary school (compared with those who did not) (OR = 1.83); Men were statistically significant less likely than women to have negative attitudes toward social support (OR = 0.72, *p* < 0.05). All respondents with negative attitudes toward social support were statistically significant more likely to be sole practitioners of veterinary medicine (OR = 1.23); to not belong to a veterinary association (OR = 1.29); to exhibit signs of serious mental health problems (OR = 1.55); to report suicidal ideation after graduating from veterinary school (OR = 1.66); and to be 40 to 59 (vs. 20 to 39) years old (OR = 1.18) (*p* < 0.05).

In their study, Kogan et al. [33] addressed the assessment of the prevalence of medical errors in the practice of veterinary medicine (near misses = NM, adverse events = AE) and the personal and professional impact on veterinarians (*N* = 606). Seventy-four percent (73.8%) of respondents reported having been involved in more than one NM (64.2%) or AE (29.5%). Following the most severe AE with which they had been involved, 42.4% felt less satisfied with their job, 37.7% felt burned out, 65 36.9% had a decrease in overall happiness, 35.1% felt that their professional reputation had been negatively impacted, 33.7% had problems sleeping, and 33.5% felt persistently guilty. Short term was defined as ≤ 1 week after the event and long term was defined as > 1 week after the event. NMs had a short-term (≤ 1 week) negative impact on professional life in 68.0% of respondents and a longer-term negative impact in 36.4%.

Mair et al. [46] used the Warwick-Edinburg Mental Wellbeing score (WEMWBS) to assess the mental wellbeing of equine veterinarians (*N* = 451) (as well as equine nurses and veterinary students) during and before the COVID-19 pandemic. The results of the 14 individual items of the WEMWBS for veterinary surgeons and veterinary nurses, and the mean total scores, were compared to the results for equine veterinary surgeons and equine veterinary nurses from the 2019 survey of the veterinary profession The mean WEMWBS score for veterinarians was 47.17 during the pandemic’ the mean score was 48.08 in a prepandemic 2019 survey [80]. There were statistically significant differences for 9 of the 14 WEMWBS items between the sums of the number of respondents who answered “never” or “rarely” and those who answered “often” or “always” for the two surveys. The two proportions test revealed statistically significant differences between the proportions of respondents who answered ”often” and ”always” for 8 items and statistically significant differences between the proportions of respondents who answered ”rarely” and ”never” for 8 WEMWBS items (there were statistically significant differences in both proportions tests for 6 items).

Mastenbroek et al. [20] tested the role of three personal resources (proactive behavior, reflective behavior, and self-efficacy) in the Job Demands-Resources (JD-R) model to predict self- and external assessment of performance of veterinarians (*N* = 860). The direct effect of job demands on self- and external assessment of performance in the role was statistically significant. Work demands were positively related to exhaustion. Exhaustion was negatively related to self- and external assessment of performance in the role.

Nett et al. [47] surveyed the prevalence of suicide risk factors, attitudes toward mental illness, and practice-related stressors among US-American veterinarians (*N* = 11.627). Nine percent of respondents suffered from severe mental health problems, and 31% of respondents had depressive episodes since completing veterinary school. At the time of the survey, 19% of respondents were receiving treatment for a mental illness. The most frequently cited practice-related stressor was the demands of practice. The mean Kessler 6 (K6) score on risk factors for suicide was 6.0, and 17% of respondents had suicidal ideation, with 1% having attemptedsuicide (since leaving veterinary school).

In a cohort study consisting of US Army medical professionals, Rivera et al. [50] examined the determination of the prevalence and relative likelihood of, among other things, mental health problems, suicidal ideation, and lack of social support among veterinarians (*N* = 101) compared with others (nontrauma physician, trauma physician, general dentist, veterinary technician, or medic). The results of logistic regression analysis showed that veterinarians were more likely to experience psychological problems than general dentists (OR = 2.53). Compared with physicians and dentists combined, veterinarians also had a higher likelihood of experiencing psychological problems (OR 1.89); sleep disturbances (OR = 2.07); and lack of social support (OR = 1.68). A total of 36.6% of veterinarians reported a lack of social support; and 52.5% reported problems falling asleep.

Schwerdtfeger et al. [13] examined the risk of suicide and depression among German veterinarians (*N* = 3,118) and compared the results with two general population samples of the same age group (mean age 41.3 years) using the Suicide Behaviors Questionnaire-Revised (SBQ-R) and the Patient Health Questionnaire (PHQ-9). Approximately twenty-eight percent (27.78%) of veterinarians were found to have depression according to the PHQ-9, of which 17.45% had moderate symptoms of depression and 10.33% had moderately severe to severe symptoms of depression. Compared to the general population, veterinarians are approximately three times more likely to have depression (OR = 0.349; 95% CI 0.309 to 0.940). Nineteen percent (19.2%) of the veterinarians studied were classified as having suicidal ideation in the past two weeks; and the majority of those patients (15.91%) reporting to have had such feelings on several days during the last two weeks; 2.31% reported almost half of the days; and 0.96% reported nearly every day in the past two weeks. Veterinarians were approximately twice as likely to express current suicidal ideation as the general population sample used (OR = 0.497; 95% CI 0.445 to 0.554). Using the SBQ-R, 32.11% of veterinarians were classified as having an increased risk of suicide (compared with 6.62% of the general population); veterinarians showed a six- to sevenfold-fold higher risk of suicide than the general population according to the SBQ-R (OR = 0.150; 95% CI 0.123 to 0.183).

Schwerdtfgeger et al. [51] published some results in the German Veterinary Journal and extended these datafrom the COPSSOQ [51]. Emotional exhaustion was more common in women (“always”: 5% or ”often”: 36%) than in men (“always”: 5% and “often” 25%, respectively). Clinically relevant depressive symptoms were identified in 27.8% of respondents.

Witte et al. [52] compared the prevalence of negative mental health outcomes among lesbian, gay, bisexual, transgender, queer, questioning, and asexual (LGBTQ +) veterinary students and veterinarians (*N* = 440) with the prevalence reported in a previous study from Nett et al. [47]. The lifetime prevalence of suicidal ideation and suicide attempts was also higher (29% of nonheterosexual cisgender individuals (cis describes a person whose gender identity is the same as their sex assigned at birth), 36% of nonheterosexual cis veterinary women, and 50% of transgender or nonbinary veterinary individuals) than those reported as comparative values from the study results of Nett et al. [47]. Transgender and nonbinary individuals had a statistically significantly higher prevalence of severe mental disorders (Kessler 6 score ≥ 13) at 41% than the comparison groups (*p* < 0.01). Nonheterosexual cis women (16%) had a statistically significantly higher prevalence of severe psychological distress than female veterinarians (*p* = 0.005). Transgender and nonbinary individuals showed the highest prevalence of previous depressive episodes (50%), which differed statistically significantly from the prevalence for male veterinarians (*p* = 0.001) and for nonheterosexual cis men (*p* = 0.01). Nonheterosexual cis women had a statistically significant (*p* = 0.003) higher prevalence of previous depressive episodes (45%) than female veterinarians in the comparison study.

## Quality assessment (EPHPP)

All studies were rated as weak; only one study was a longitudinal study (Rivera et al. 2021) [50], and there was no blinding. Due to the study types, there were also weaknesses in selection bias. The majority of studies considered confounders, so a strong rating could be assigned for this aspect [13, 21, 36–38, 41, 43–45, 47, 48, 50, 52]. The study drop-out item was not applicable to any of the studies. Evidence from the included studies is not indicated.

## Discussion

In this section, the current scoping review is summarized on the basis of the search results, and then, the main findings are compared to similar research articles. The limitations of this scoping review are also described. Finally, recommendations for practice are presented. The present scoping review aimed to summarize studies on psychological workload and its possible related health consequences in veterinarians. The results of the studies presented here indicate a very wide variety of mental health outcomes within the veterinary profession (for example, depression, burnout, anxiety, and suicidal risk factors). Therefore, despite a broad search strategy, it is possible that not all studies appropriate for this review were identified. Furthermore, the search could have been expanded to include terms related to occupational health (for example, compassion fatigue). Nevertheless, 21 studies (plus seven additional studies without a standardized questionnaire) from nine countries (England, USA, Canada, Australia, New Zealand, Germany, Finland, Turkey, Netherlands) were found. This again highlights the increasing number of publications and related research on the topics of suicide, burnout, and depression among veterinarians, as described by Brysk et al. [24]. The studies were assessed according to EPHPP and were classified as weak according to the existing guidelines. Although the EPHPP evaluation tool is suitable not only for randomized controlled trials or controlled clinical trials but also for cohort or other studies, only weak study qualities were achieved, as expected. Therefore, the need for further studies, such as intervention studies investigating the effect of preventive measures, arises here as well.

The results of the studies indicate an increased prevalence of psychological stress factors and conditions among veterinarians, highlighting risk factors for mental health, such as burnout, anxiety and depressive disorders. Taking a gender-specific view of the studies presented, female veterinarians tend to have poorer mental health than male veterinarians. The higher proportion of women in veterinary medicine should be taken into account [81]. Epidemiological studies in recent years have found that the prevalence of depression is generally higher in women than in men [82]. The comorbidity of anxiety and depression was identified as a risk factor for suicide in veterinarians by Nett et al. [47]. In Shirangi [49], longer work hours were associated with increased anxiety and depression in female veterinarians overall and subdivided by women with and without children. Similarly, the aforementioned review by Platt and colleagues [11] indicates that female veterinarians are most at risk for negative stress outcomes such as suicidal ideation, mental health problems, and job dissatisfaction. In this respect, work-life balance among female veterinarians should be focused on at the organizational level. Hatch et al. [44] infer from their data that increased anxiety occurs in employees who have practiced veterinary medicine for 10 to 15 years. This is explained by the association of increasing responsibilities in the practice but also with increasing family responsibilities, which are often assumed by female veterinarians. These include, among other things, the reconciliation of working hours and family. Longer working hours were associated with increased anxiety and depression in Shirangi’s study of female veterinarians both with and without children (Shirangi et al. 2013) [49]. Findings related to the LGBTQ + population by Witte et al. (2020) suggest a higher likelihood of negative mental health outcomes than among veterinarians in general. Within the LGBTQ + respondent sample, transgender and nonbinary individuals have the highest risk of negative mental health outcomes, and nonheterosexual cis men have the lowest risk [52]. Further research is recommended to ensure that LGBTQ + affiliated veterinarians receive appropriate support.

The MBI (including the MBI-HSS) questionnaire (Maslach and Jackson 1981) [83] was used four times as the most common survey instrument in the studies presented. In a study conducted by Heath [84] among veterinary students, burnout as assessed by the MBI decreases with increasing years after veterinary school but still exceeds 25% of the reference data for personal burnout compared to work-related burnout, which approaches the reference values (reference baseline data are from the 2001 ABS Health Survey of the Australian population [85]) (Hatch et al. 2011) [44]. That burnout decreases with increasing years after graduation has also been described by Reijula et al. [36] in their study. There, too, in the estimation the overall prevalence of work-related burnout (with the MBI), 40% were found to have moderate symptoms and 1.7% severe symptoms. The classification of burnout risk according to the three MBI subscales (according to Maslach) showed an 8.8% high risk in a study with hospital doctors. Another 11.8% of the participating doctors showed a moderate risk of burnout [86]. Similar results were found for the burnout prevalence of anesthesiologists [87]. This implies a higher risk of burnout than in human medical professions.

The concept of resilience is becoming increasingly important in relation to protective health factors [88], as people with high resilience show fewer burnout symptoms and fewer consequences of mental and psychosomatic illnesses [89]. The results of the CD-RISC scale, which was developed by Best et al. [39], indicate that the scores of 74.5% of the veterinarians studied are below the scale’s (general population of the United States) comparison norm of 80.7% [90]., Mental and physical health emerged as strong predictors of resilience among veterinarians in the study by Perret et al. [48]. Considering that veterinarians are also exposed to enormous physical hazards [91, 92] and have a high incidence rate of occupational injuries, which is more than double that of doctors of human medicine [93], prevention and support services should address both mental and physical health.

In Fritschi et al. [37], participants reported increased levels of work stress and distress due to working hours. The veterinarians studied by Reijula et al. [36] considered a reduction in working hours or a reduction in on-call duty to be suitable means of reducing stress. A major stress factor among veterinarians is working time. In an older study (survey from 1999, therefore not included as part of this review) by Gardner et al. [18], working hours were considered to be one of the main stress factors in veterinary practice.

Depression is generally among the most frequently cited risk factors for suicidal behaviors [94] and is frequently referenced in risk factor guidelines of national and international organizations and included in structured suicide risk assessments [95]. In a qualitative study by Waters [96], small animal veterinarians in the greater Seattle area were interviewed about their professional experiences related to depression, suicidality, and coping. Analysis of the interviews revealed that attachment loss and trauma were the most important factors contributing to depression and suicidality within the veterinary profession (Waters et al. 2019). A study by Schwerdtfeger [13, 51] was the first to compare German veterinarians with the German general population in terms of depression, suicide risk, and suicidal ideation. The analyses indicate that veterinarians have a statistically significant increased risk for depression and suicidal ideation compared to the general population in Germany. The results of the review indicate a disproportionate incidence of depression among veterinarians. This indicates the urgency of implementing appropriate measures and interventions to reduce depression and suicidality among veterinarians.

## Conclusion

These findings of our scoping review highlight the importance of reducing psychological stressors to increase the overall mental wellbeing of this population group and improve the mental wellbeing of veterinarians. There is a need for further studies, such as intervention studies investigating the effect of preventive measures.

Strategies for coping with work-related stress in veterinarians should be developed in a timely manner. This includes gender-differentiated strategies to offer female veterinarians adequate methods of organizing work routines as well as reinforcement of social support. Furthermore, it is necessary to identify concrete work stresses in further surveys. For this purpose, further research is necessary that includes the requirements of emergency and on-call services as additional potential stress factors. Coaching and counseling on communication and conflict management, as well as courses/seminars on stress management, may be helpful to better learn how to manage stress, especially after critical deployments (for example, complex operations, complicated cases of illness, potential for conflict among pet owners). Support services such as counseling centers and mentoring programmes for early career professionals should also be made available.

Interventions aimed at the veterinary profession have been described by Bartram et al. (2010), which can be well derived from the results we have described [97]. The authors refer to areas such as mental health promotion (e.g., mental health education initiatives integrated into the curriculum), monitoring of trends (e.g., implementation and monitoring of interventions), accessible and appropriate support services (e.g., introduction of a telephone counseling service), other personal and work-related stressors (for example, training to improve communication skills), work-home interaction (for example, forming on-call collaborations with local practices), or future research (for example, mixed methods or qualitative interviews with individuals experiencing suicidal ideation to perceive barriers to seeking help).

Professional organizations and veterinary schools should provide training on managing work-related anxiety and depression, as well as resilience-building programmes to improve the mental wellbeing of veterinarians and potentially reduce turnover in this profession. According to the American Veterinary Medical Association’s (AVMA) 2020 Veterinary Census report, poor work-life balance is the top reason to leave the veterinary profession: encourage parental support or hire relief vets to balance workload.

Several strategies can create a better work environment, improve employee retention and boost morale and wellbeing. Platforms such as the Mind Matters Initiative (MMI), which aims to improve the mental health and wellbeing of veterinary staff (including veterinary surgeons), can be helpful for this [98].

## Data Availability

Not applicable.

## Abbreviations

PRISMA: Preferred Reporting Items for Systematic Reviews and Meta-Analyses
EPHPP: Effective Public Health Practice Project tool
HADS: Hospital anxiety and depression scale
HADS-A: Hospital anxiety and depression scale—Anxiety
HADS-D: Hospital anxiety and depression scale—Depression
MBI: Maslach Burnout Inventory
DCSQ: Swedish Demand-Control-Support Questionnaire
PSS: Perceived Stress Scale
ProQOL: Professional Quality Of Life Scale
CD-RISC: Connor-Davidson Resilience Scale
NEO-FFI: NEO Five-Factor Inventory
JSS: Job Stress Survey
CFSS: Compassion Fatigue Short Scale
K6/K10: Kessler Psychological Distress Scale
CBI: Copenhagen Burnout Inventory
DASS: Depression Anxiety and Stress Scale
GHQ: General Health Questionnaire
CGHQ: Chronicity and the General Health Questionnaire
PA: Positive Affect
NA: Negative Affect
PNOSI: Positive and Negative Occupational Stress Inventory
WHI: Work-Home Interference
NM: Near misses
AE: Adverse events
WEMWBS: Warwick-Edinburg Mental Wellbeing score
JD-R: Job Demands-Resources model
SBQ-R: Suicide Behaviours Questionnaire-Revised
PHQ-9: Patient Health Questionnaire
COPSSOQ: Copenhagen Psychosocial Questionnaire
HADS-T: Hospital anxiety and depression scale - Total
WHI_N: Negative Work-Home-Interaction
WHI_P: Positive Work-Home-Interaction
